# Efficacy of *Anystis baccarum* against Foxglove Aphids, *Aulacorthum solani*, in Laboratory and Small-Scale Greenhouse Trials

**DOI:** 10.3390/insects12080709

**Published:** 2021-08-07

**Authors:** Taro Saito, Michael Brownbridge

**Affiliations:** 1 Vineland Research and Innovation Centre, 4890 Victoria Avenue North, Box 4000, Vineland Station, ON L0R 2E0, Canada; 2 BioWorks Inc., 100 Rawson Road, Victor, NY 14564, USA; mbrownbridge@bioworksinc.com

**Keywords:** predatory mite, generalist predator, *Anystis baccarum*, biological control

## Abstract

**Simple Summary:**

The foxglove aphid (FGA), *Aulacorthum solani* (Keltenbach), feeds on many important greenhouse crops in Canada. Currently, biological control of this pest is difficult as existing biocontrol agents are only moderately effective. Access to a generalist predator would strengthen biocontrol programs for this and other similarly challenging pests. *Anystis baccarum* (L.) is a globally distributed generalist predatory mite. We assessed the predatory efficacy of *A. baccarum* against FGA in a series of laboratory and small-scale greenhouse trials. The laboratory trials showed that *A. baccarum* readily consumed all FGA life stages and was particularly effective against first instars. In a greenhouse trial on sweet peppers, working together with *Aphidius ervi* Haliday, an aphid-specific parasitoid wasp, *A. baccarum* successfully eradicated the aphids and higher fruit yields were obtained compared to plants protected by the parasitoids only. Pepper plants also became naturally infested with western flower thrips during the trial, which caused feeding damage to the fruits. The fruits were likely to have less thrips’ feeding damage on plants carrying *A. baccarum* as the predator also fed on these pests. The results demonstrate that *A. baccarum* could be a useful addition to greenhouse IPM programs for hard-to-control pests like FGA, especially when they occur together with other pest species.

**Abstract:**

A generalist predatory mite, *Anystis baccarum* (L.), has been identified as a key predator of small, soft-bodied pest species in various agroecosystems around the world. The foxglove aphid *Aulacorthum solani* (Keltenbach) is a new problematic pest in Canadian greenhouses. Laboratory colonies of *A. baccarum* were established and its predatory efficacy against *A. solani* was assessed. In laboratory trials, *A. baccarum* ate approximately one adult aphid or seven first instar aphids in 24 h. In a greenhouse bench trial on sweet peppers with the free-flying aphid parasitoid, *Aphidius ervi* Haliday, the population dynamics of *A. solani* in the presence or absence of *A. baccarum* was evaluated. Although the parasitoid alone successfully eradicated *A. solani*, when *A. baccarum* were present on the plants, the aphid population was eradicated more rapidly. Fruit yield was also 15% higher from plants where *A. baccarum* was released than the control (without *A. baccarum*). Furthermore, plants were naturally infested by *Frankliniella occidentalis* (Pergande) during the trial, which caused visible feeding damage to the fruits. *Anystis baccarum* also predates on thrips and thrips’ feeding damage to the fruits was reduced on plants where *A. baccarum* was released. *Anystis baccarum* was able to establish in sweet peppers and was determined to be complementary to the current practice of using *A. ervi* for the biological control of *A. solani*.

## 1. Introduction

Interest in the generalist predator *Anystis baccarum* (L.) (Acari: Anystidae) in agroecosystems has increased in recent years. For example, in UK apple orchards, *A. baccarum* has been credited with providing natural control of European fruit tree red spider mites, *Panonychus ulmi* (Koch) (Acari: Tetranychidae), and measures to promote a conservation biocontrol strategy to enhance the prevalence and impact of the predator have been described [1,2]. In organic potato crops in New Zealand, abundant *A. baccarum* populations developed in response to heavy infestations of the invasive potato–tomato psyllid, *Bactericera cockerelli* (Šulc) (Hemiptera: Triozidae), and presented a potential solution for suppression of the pest [3]. In tea plantations in China, *A. baccarum* has been identified as a key natural predator of the tea green leaf hopper, *Empoasca onukii* Matsuda (Hemiptera: Cicadallidae), especially in rows intercropped with Bahia grass (*Paspalum notatum* Flügge, Poaceae) compared to those with natural ground cover only [4]. In spite of its near global distribution and voracious predatory behaviour, *A. baccarum* has not generally been considered for use in inundative control strategies. In large part, this has been due to a lack of efficient mass-rearing methods for the predator, which has deterred commercial investment in its development as a biological control agent.

In Canada, several new pests have become established in greenhouse ornamentals and vegetables in recent years. The lack of effective biocontrol agents for some of these means that growers have had to revert to using chemical insecticides, disrupting established biological control programs in the process. One such pest is the foxglove aphid (FGA), *Aulacorthum solani* (Keltenbach) (Hemiptera: Aphididae), which feeds on numerous plant species including many important ornamental and vegetable crops. Its rise in pest status in Canada may be due in part to the widespread adoption of biocontrol strategies and the concurrent decline in the use of broad-spectrum pesticide sprays [5,6]. Commercially-available aphid parasitoids have typically provided inconsistent control or have not been sufficiently efficacious to be considered effective stand-alone treatments [7]. Access to a generalist predator could provide a strong foundation for a biocontrol program for this and other challenging pests that lack specialist natural enemies [8]. For example, biological control of the tomato leafminer, *Tuta absoluta* (Meyrick) (Lepidoptera: Gelechiidae), in European vegetable greenhouses became possible with the introduction of the generalist predatory bug, *Macrolophus pygmaeus* Rambur (Hemiptera: Miridae) [9]. However, *M. pygmaeus* is not native to North America and cannot simply be imported as a possible solution for FGA. This has prompted the search for native generalist predators that could underpin biocontrol programs for FGA and other pests in protected growing systems, and has reignited interest in the potential use of *A. baccarum*.

Recent work by Saito and Brownbridge [10] demonstrated the predatory capacity of *A. baccarum* against greenhouse pests. This was enabled by the successful development of a rudimentary mass-rearing system. Here, we describe a series of trials designed to further evaluate the performance of *A. baccarum* as a greenhouse biocontrol agent and assess its efficacy against FGA.

## 2. Materials and Methods

### 2.1. Rearing of Pest Insects

A colony of FGA was established on potted pansies, *Viola tricolor* L. (Violaceae), using adults acquired from infested Peruvian lilies, *Alstroemeria psittacina* Lehmann (Alstroemeriaceae). The colony was maintained in thrips-proof screened dome-shaped cages (BugDorm—2120F, MegaView; Science Co., Ltd., Taichung, Taiwan) placed in a walk-in growth chamber (23 ± 1 °C, 60% RH,d and 16:8 L:D). A mixed-aged aphid population was maintained on the plants and individuals were collected directly from the cages using an aspirator when required. A second colony of FGA was established on sweet peppers, *Capsicum annuum* L. (Solanaceae) (var. Currier, Stokes Seeds, Thorold, ON, Canada), and maintained in the same manner as the colony on the pansies. Having colonies on both hosts allowed trials to be done on representative ornamental and vegetable crops.

### 2.2. Predators

*Anystis baccarum* specimens were originally collected in the backyard of a residence in St. Catharines, Ontario, Canada, in 2011. The original colony (colony 1) was maintained in thrips-proof screened dome-shaped cages on potted chrysanthemums, *Chrysanthemum indicum* L. (Asteraceae) (var. Brighton or Chesapeake, Syngenta Flowers North America, Gilroy, CA, USA) that were artificially infested with western flower thrips, *Frankiliniella occidentalis* (Pergande) (Thysanoptera: Thripidae). Another colony (colony 2) was established from mites that invaded our FGA colony and has been maintained in the same type of dome-shaped cages on potted chrysanthemums infested with FGA. Note that *A. baccarum* populations are comprised solely of females that reproduce parthenogenetically. Both colonies were maintained in a walk-in growth chamber (25 °C: 20 °C L:D, 60% RH, and 16:8 L:D). A third colony was established using the same set up but the host plants were infested with a mix of western flower thrips, FGA, and bran mites, *Tyrophagus putrescentiae* (Schrank) (Acari: Acaridae). This colony is referred to as the generalized colony.

### 2.3. Experimental Designs

#### 2.3.1. Laboratory Cup Trial: Efficacy against Aphids of Mixed Age

Three treatments were tested: (1) untreated control; (2) *A. baccarum* from colony 1; and (3) *A. baccarum* from colony 2. Aphids of mixed ages ranging from third instars to adults were collected from the aphid colony maintained on pansies. Ten individuals were placed in a small plastic cup (opening 6 cm diameter, bottom 4 cm diameter, height 3 cm, volume 2 oz, Solo^®^ P200N, Dart Container Corporation, Mason, MI, USA) with a vent hole in the lid (covered with a 2 cm diameter thrips-proof mesh screen) containing a clean pansy leaf and some moist paper towel. For the *A. baccarum* treatments, one adult mite was introduced into each cup. These cups were transferred to a plastic storage bin (60 × 40 × 15 cm, L × W × H) that was held in a walk-in growth chamber (25 °C:20 °C L:D, 60% RH, and 16:8 L:D). The number of surviving aphids in each cup was recorded 24 h and 72 h later. If mites were missing or dead in the *A. baccarum* treatment cups, the data were excluded from the analysis. The trial was repeated three times over time, each trial having *n* = 10 per treatment. Each treatment had a total of *n =* 30, *n =* 26, and *n =* 26 replicates (cups) for the control, ‘colony 1′ *A. baccarum*, and ‘colony 2′ *A. baccarum* treatments, respectively. Data collected after 24 h and 72 h were square-root transformed and analyzed by a repeated measures ANOVA model using the mixed procedure of SAS (Proc MIXED, SAS release 9.4, SAS Institute Inc., Cary, NC, USA). In this model, the treatment, time, and their interaction were considered as fixed factors, whereas the repetition block and block*treatment were considered as random factors. An AR(1) covariance structure was also chosen to consider the dependency between observations taken over time on the same cup nested with (block*treatment). Tukey’s multiple comparison was used to contrast the results.

#### 2.3.2. Laboratory Cup Trial: Efficacy against 1st Instar Aphids

The experimental set-up was similar to that as described above except that all of the aphids were in the same developmental stage and were of similar age (first instar, 0–20 h old). When 1st instar nymphs were needed, adult aphids were collected from the colony on pansies and placed in the small vented plastic cups containing a pansy leaf on a moist paper towel. The plastic cups were held in a growth chamber at 23 ± 1 °C, 60% RH and 16:8 L:D. The 1st instar nymphs were removed from the cups 24 h later. Per the protocols described in 2.3.1, ten first instars were placed into each assay cup and survivors were counted 24 h after introduction of the predatory mites. The trial was repeated three times over time, each trial having *n =* 10 per treatment. Each treatment had a total of *n =* 30, *n =* 29, and *n =* 30 replicates for the control, ‘colony 1′ *A. baccarum*, and ‘colony 2′ *A. baccarum*, respectively. Data were square-root transformed and analyzed by a linear mixed model using Proc MIXED with the treatments as a fixed factor, and the trial repetition block and its interaction with the treatments as the random factors. Tukey’s multiple comparison was used to contrast the results.

#### 2.3.3. Laboratory Cage Trial: Effects on Aphid Population Growth on Sweet Pepper

The ability of *A. baccarum* to suppress aphid population growth was assessed using cages constructed from clear plastic bottles (2.0 L, commonly used for carbonated beverages). The top third of the bottle was cut off to make a cylinder with a bottom. Two ventilation holes (7 cm diameter) were made on opposite sides of the wall and were covered with thrips-proof mesh. Sweet pepper plants (var. Morraine, De Ruiter ^®^ seeds Canada, Leamington, ON, Canada) were grown in rockwool strips in the greenhouse. When the plants’ second set of true leaves were fully developed, they were individually transplanted in to 6” pots on the day of the experiment. A total of five apterate adult FGA were collected from the colony, maintained on sweet peppers, and released onto each plant. Adult *A. baccarum* were placed onto half of the infested plants, one mite per plant, and no mites were placed on the remaining plants (control). The mites used in this experiment were collected from the generalized colony. The plastic bottle cages were then placed over the plants with 1 cm of the bottom edge (cut-off edge) inserted into the growing medium, thereby preventing the mites from escaping. The plants were kept in a growth chamber (25 °C:20 °C L:D, 60% RH, and 16:8 L:D) for 72 h and surviving aphids were counted; survivors were assigned to one of three categories (alate adults, apterate adults, and nymphs) and the total population determined. Each trial contained seven plants per treatment and was repeated three times over (total *n =* 21 per treatment). Data for each of the aphid categories were analyzed separately by a linear mixed model using Proc MIXED with the treatments as a fixed factor, and the trial repetition block and its interaction with the treatments as the random factors. The data for aphid alates, apterates, and nymphs were square-root transformed and total aphid population data were log-transformed prior to the analysis. 

#### 2.3.4. Greenhouse Trial: Effects on Foxglove Aphid Population Dynamics on Sweet Pepper 

The assay was conducted on sweet pepper grown in coco-coir growbags (Milleniumsoils coir™, Vgrove Inc, St. Catharines, ON, Canada). The preliminary trial carried out using potted peppers on an open greenhouse bench showed that *A. baccarum* alone was not able to suppress FGA population growth to acceptable levels. FGA populations grew from 3 to 100 aphids per plant in three weeks in the presence of *A. baccarum*, although it was significantly better than the untreated control for which the aphid population exceeded 500 over the same time period. The current treatment combined the predatory action of *A. baccarum*, a generalist, with the specialist *Aphidius ervi* Haliday (Hymenoptera: Braconidae), an aphid-specific parasitoid wasp commonly employed for the control of FGA in commercial greenhouses [11]. Sweet pepper plants (var. Morraine) were grown according to common commercial practice with some modifications. Peppers were seeded in a rockwool germination sheet and kept in a growth chamber (28 ± 1 °C, 16 L:8D) for two weeks. They were then transplanted into rockwool blocks (Grodan^®^ Delta, Grodan North America, Milton, ON, Canada) and held on a flood bench for four more weeks until the roots had grown through the bottom of the blocks. The six-week-old plants were placed onto coco growbags that had been pre-saturated with fertilized water. One coco growbag held 3 pepper plants, which was considered as one replicate, with a total of 5 replicates per treatment. Three adult FGA were inoculated onto each plant (3 plants per replicate = a total of 9 aphids per replicate) and left undisturbed for 72 h before *A. baccarum* and *A. ervi* were released. As the parasitoids could not be confined to individual treatment blocks, they were released into the entire greenhouse at a rate of two *A. ervi* females/m^2^. Adult *A. baccarum* were placed onto half of the replicates, one mite per plant, and no mites were placed on the remaining five replicates (control). Upon receipt, *A. ervi*-parasitized aphid mummies were placed in a cage and adults were allowed to emerge and mate for 48 h; adults were provided with honey–water as a food source. After 48 h, adults were collected in 6 cylindrical containers, each containing 26 females and 5 males. As the coco growbags (five per treatment, ten in total) were randomly placed on two benches (five per bench), the parasitoids were released by placing one container at each of the six points within the crop, three places per bench (the middle and both ends of a bench). The adult *A. ervi* were thus free to choose which plants they visited and the aphid patches they parasitized. One adult female *A. baccarum* was released onto each pepper plant (three adult females per replicate, obtained from the generalized colony). The plant foliage was touching within a replicate so that aphids and *A. baccarum* were able to move freely among the three plants. In an effort to contain *A. baccarum* within a replicate, the bench surface on which the coco growbags were placed was covered with landscape fabric and labelling tape was used to create a border around each replicate (10 cm away from the sides of the coco growbag); petroleum jelly was applied to the tape and irrigation lines to create a barrier that restricted movement of the predatory mites outside of the replicate boundary. The total number of FGA and aphid mummies, i.e., *A. ervi* parasitized aphids, were counted on all three plants within each replicate at T = 0 (pre-treatment), 1 week, 2 weeks, 3 weeks, 4 weeks, 5 weeks, 6 weeks, 7 weeks, and 8 weeks after release of the natural enemies. As *A. baccarum* tends to hide during the warmest part of the day and gravid females are not generally found on the foliage, the numbers recorded during the trial were likely inaccurate; however, the timing of the appearance of nymphs on the foliage was accurately noted due to the petroleum jelly barrier trapping new-generation *A. baccarum* nymphs and larvae (not included in the data). Western flower thrips also naturally occurred on the plants during the trial. Pepper fruits began to mature at T = 10 weeks and thrips’ feeding scars were often visible. Consequently, thrips’ feeding damage assessments were done on fruits harvested at T = 10 weeks, 11 weeks, and 12 weeks using a visual rating scale of 0 to 5, where 0 = no damage, 1 = less than 10% of the surface area scarred, 2 = less than 30%, 3 = less than 50%, 4 = less than 70%, and 5 = more than 70%). 

FGA (total number per replicate of three plants, *n =* 5 for each treatment) were square-root transformed and analyzed by a repeated measure ANOVA model using Proc MIXED. In this model, the treatment, time, and their interaction were considered as fixed factors, whereas the benches were considered as a random factor. Furthermore, an AR(1) covariance structure was also chosen among many structures based on the AIC criterion to consider the dependency between observations taken over time on the same replicate nested with (bench*treatment). Aphid mummy data were excluded from the comparison as the parasitoid was free-flying in the greenhouse. The number of mature fruits harvested was aggregated per replicate and the mean thrips’ feeding damage score per fruit was calculated per replicate. These data were analyzed by a randomized block ANOVA model using Proc MIXED with the treatments as a fixed factor and the benches (or blocks) as a random factor.

## 3. Results

### 3.1. Laboratory Cup Trial: Efficacy against Aphids of Mixed-Age 

**Figure 1:**
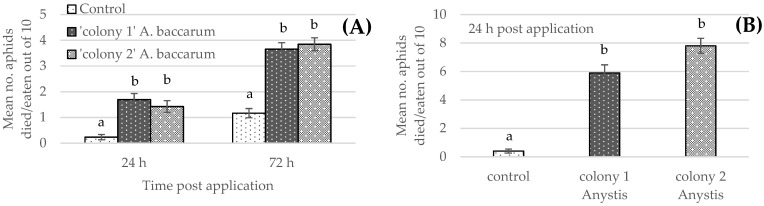
Mean mortality (number of dead aphids out of original 10) ± SE of mixed foxglove aphid (FGA) life stages presented to the predatory mite *Anystis baccarum*, in laboratory cup trials: (**A**) mean number of dead/eaten aphids out of 10 late instar nymphs and adult FGA, and (**B**) mean number of dead/eaten aphids out of 10 first instar FGA. Assigned lowercase letters indicate the results from Tukey’s test; data points with different letters indicate that values are significantly different from the others.

The analysis showed that *A. baccarum* from both colonies caused significantly higher mortality of fully grown adult or late instar FGA compared to the untreated control at both 24 h and 72 h observation (F _2, 4_ = 68.20, *p* = 0.0008, Figure 1A). The aphid mortality was significantly higher at 72 h than it was at 24 h, regardless of the treatments (F _1, 152_ = 167.04, *p* < 0.001). Differences in feeding efficacy between the two groups of mites were not significant (Figure 1A). Note that there were no significant block effects from the trial repetition and no interaction with the treatments. The mean number of FGA eaten by *A. baccarum* in 24 h was 1.69 ± 0.24 and 1.42 ± 0.25 for mites from colony 1 (reared on thrips) and colony 2 (reared on FGA), respectively. In 72 h, the number of FGA eaten was 3.65 ± 0.23 and 3.85 ± 0.25 for *A. baccarum* from colony 1 and colony 2, respectively. It appears that one adult or late instar FGA sates the appetite of adult *A. baccarum* for 24 h. 

### 3.2. Laboratory Cup Trial: Efficacy against 1st Instar Aphids

*Anystis baccarum* consumed 1st instar FGA more rapidly than older instars or adults (see Section 3.1). The trial was concluded after 24 h as the majority of the aphids had been consumed. The mean number of 1st instar FGA eaten by *A. baccarum* in 24 h was 6.1 ± 0.57 and 7.8 ± 0.52 for mites from colony 1 (reared on thrips) and colony 2 (reared on FGA), respectively. The analysis showed that *A. baccarum* from both colonies caused significantly higher mortality of 1st instar FGA compared to the untreated control (F _2, 4_ = 159.83, *p* = 0.0002, Figure 1B). Differences in feeding efficacy between the two groups of mites were not significant (Figure 1B); however, among 30 replicates per treatment, 14 of the ‘colony 2’ mites consumed all ten FGA, whereas only six of the ‘colony 1’ mites consumed all ten FGA.

### 3.3. Laboratory Cage Trial: Effects on Aphid Population Growth

**Figure 2:**
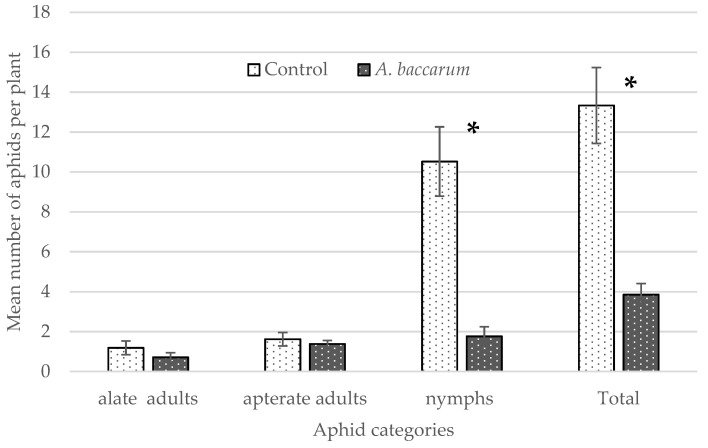
The mean number ± SE of foxglove aphids of different developmental stages on a pepper plant in the laboratory cage trial 72 h after five apterate adult aphids were released onto the plants. Note that * indicates statistically significant differences in values by treatment, i.e., control vs. *Anystis baccarum*.

The mean number of alate and apterate adult aphids did not differ between the untreated control and the *A. baccarum* treatment (Figure 2; alates F _1, 2_ = 0.94, *p* = 0.4340; apterates F _1, 2_ = 0.01, *p* = 0.9881). However, the mean number of aphid nymphs was significantly lower in the *A. baccarum* treatment (F _1, 2_ = 28.54, *p* = 0.0333), contributing to the significant difference detected between the total number of aphids in both treatments (F _1, 2_ = 35.97, *p* = 0.0267). 

### 3.4. Greenhouse Trial: Combined Efficacy of the Aphid Parasitoid Aphidius ervi and Anystis baccarum against Foxglove Aphids on Sweet Pepper

**Figure 3:**
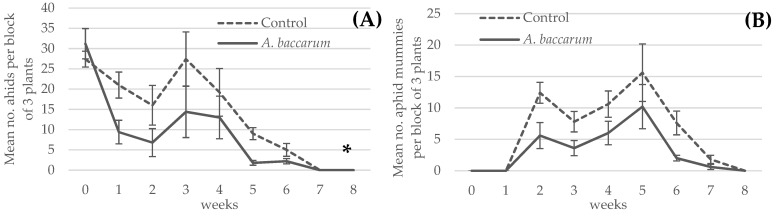
(**A**) Mean foxglove aphid population ± SE per block of three pepper plants in the greenhouse trial. An assigned asterisk indicates overall significant difference between the treatments. (**B**) Mean aphid mummy population ± SE per block of three pepper plants in the same trial. Note that no statistical analysis was applied to (**B**) due to the interdependency of the parasitoid activity (producing the aphid mummies) among the replicates and treatments.

Results of the FGA population data (Figure 3A) showed that both treatment and time had a significant impact on aphid numbers (F _1, 7_ = 10.07, *p* = 0.0156 for treatment factor; F _7, 56_ =27.05, *p* < 0.0001 for time factor) but their interaction was not significant (F _7, 56_ = 1.95, *p* = 0.0783). In both treatments, FGA populations significantly declined over time and were completely eliminated by T = 7 weeks post treatment (Figure 3A). However, the plants with *A. baccarum* treatment had a significantly lower aphid population, which may be attributed to predation on adult FGA by adult *A. baccarum* during the first week. 

The number of aphid mummies in the two treatments (Figure 3B) showed that the dynamics were similar in both treatments. Aphid mummies were first observed at T = 2 weeks and increased until T = 5 weeks, after which time FGA hosts were not readily available (Figure 3A). There were more aphid mummies observed in the control treatment, likely because of the relative availability of more aphid hosts in the control treatment, which allowed higher rates of parasitism to occur. In Figure 3A, a brief increase in aphid numbers was observed in both treatments between T = 2 and T = 3 weeks, which was probably due to the relative absence of adult *A. ervi* in the crop at that time. Figure 3B shows that the number of aphid mummies decreased during that period, reflecting the lower population of adult *A. ervi* on the plants at that time. The similarity in the observed dynamics is likely caused by the interdependency between the treatments for the parasitoid. However, the more rapid decline in FGA numbers in the *A. baccarum* treatment during the first week and fourth week (Figure 3A) is likely attributed to *A. baccarum*. Although there was only one *A. baccarum* observed at T = 2 and 3 weeks combined, the second generation of *A. baccarum* as newly hatched six-legged larvae was confirmed at T = 4 weeks and the third-generation larvae were beginning to appear from T = 7 weeks. Despite a lack of confidence in the accuracy, there was a mean of 1.8 ± 0.3 *A. baccarum* observed per three plants grown in a coco growbag at T = 8 weeks and it increased to 5.4 ± 0.8 *A. baccarum* by T = 12 weeks.

**Figure 4:**
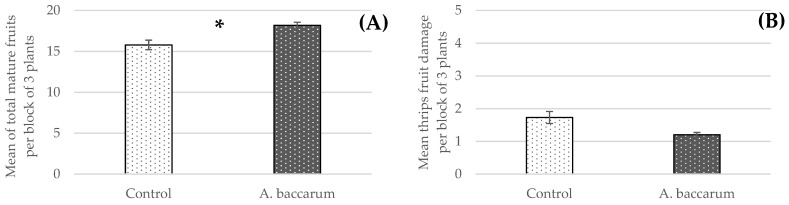
Fruit yield data obtained from the greenhouse trial. (**A**) Mean ± SE total number of harvested mature pepper fruits per block of three plants with assigned asterisk indicating a significant difference in yield between treatments. (**B**) Mean thrips’ feeding damage per fruit, aggregated per block of three plants ± SE. Score rating (*y*-axis): 0 = no feeding damage, 1 = less than 10% surface area, 2 = less than 30%, 3 = less than 50%, 4 = less than 70%, and 5 = more than 70%.

The mean total yield of mature fruits was significantly higher in the *A. baccarum* treatment than in the control treatment (F _1, 7_ = 12.00, *p* = 0.0105, Figure 4A). Feeding damage caused by western flower thrips was observed from T = 4 weeks. The thrips’ population increased over time and their feeding damage was visible on some fruits. ANOVA showed that the fruits in the *A. baccarum* treatment had a high tendency to have less thrips’ feeding damage than fruits in the control treatment (F _1, 7_ = 5.15, *p* = 0.0575, Figure 4B). 

## 4. Discussion

**Figure 5:**
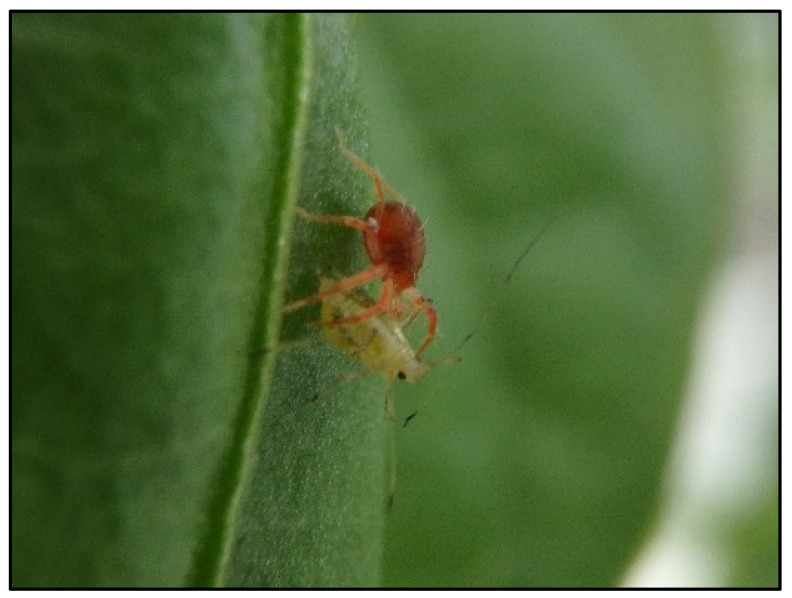
*Anystis baccarum* adult consuming an adult foxglove aphid in the greenhouse trial.

The series of laboratory trials not only showed that *A. baccarum* is an efficient predator of FGA but also revealed interesting characteristics regarding the predation efficacy of *A. baccarum* against the aphid. The first characteristic is host fidelity or rather prey fidelity in this case. In the laboratory efficacy tests against different life stages of FGA (prey-stage), two groups of *A. baccarum* were included in the trial: ‘colony 1’ mites were reared solely on western flower thrips and had never been exposed to aphids prior to the trial; and ‘colony 2’ mites were reared exclusively on FGA for several generations. Although *A. baccarum* is known to have a wide host range [2,4], if the degree of its prey fidelity was high, ‘colony 2’ mites should have shown greater efficacy against FGA than ‘colony 1’ mites. When tested against older nymphs and adult FGA, however, there was no observable difference in efficacy between the two groups of mites. When tested against 1st instar FGA, although ‘colony 2’ mites consumed more 1st instar FGA than ‘colony 1’ mites, differences in aphid consumption were not statistically significant. As a result, *A. baccarum* appears to be a truly opportunistic generalist with a low degree of prey fidelity. The second characteristic is the prey-stage preference. In the laboratory cage trial against FGA, it was clear that *A. baccarum* preferentially feeds on immature stages of the aphid. The smaller body size of FGA nymphs likely makes it easier for *A. baccarum* to capture and consume them. Similar findings have been reported in other generalist predatory mites against two-spotted spider mites, *Tetranychus urticae* Koch (Acari: Tetranychidae). The prey consumption rate was inversely related to prey size and the predatory mites preferentially fed on eggs and larval stages of *T. urtricae* over older, larger stages [12,13]. From observations of *A. baccarum* behaviour in the rearing colony, when there is an abundance of aphids available, the mites ignore older and larger aphids (an *A. baccarum* adult is similar in size to an FGA adult, Figure 5); however, when aphids are scarce and the mites are more actively searching for prey, the mites are more aggressive and actively engage with and subdue adult FGA.

**Figure 6:**
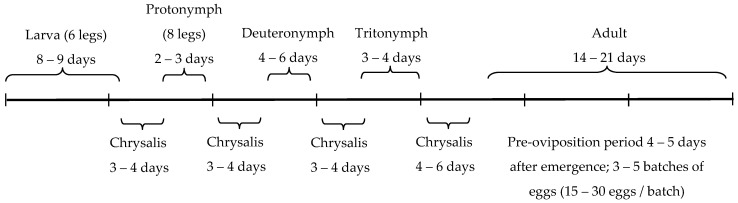
Life cycle of *Anystis baccarum* as observed in a laboratory colony (25 °C: 20 °C L:D, 60% RH, and 16:8 L:D, T. Saito, personal observations). Note the line scaling is in weekly increments.

Nonetheless, the aphid consumption rate of *A. baccarum* is not as notable as that of other aphidophagus predators. Ladybeetles and lacewings, for example, can consume 5 to 20 aphids per day depending on the life stage of the predator [14,15,16]. In the preliminary open bench trial in a greenhouse using sweet peppers and FGA, it was determined that *A. baccarum* alone, at the release rates used, cannot suppress FGA population growth. To compensate for the relatively low aphid consumption rate, the abundance of *A. baccarum* could be increased or a combined application with another biological control agent could be considered. The idea of using a generalist and a specialist parasitoid for aphid control is increasingly supported by results from other trials. In apple orchards, control of woolly apple aphid was improved when a community of generalist predators was present with the specialist parasitoid *Aphelinus mali* (Haldeman) (Hymenoptera: Aphididae) compared to control provided by the parasitoid alone [17]. Similarly, Snyder et al. [18] found that in spite of observed intraguild predation (IGP) of the parasitoid *Aphelinus asychis* Walker (Hymenoptera: Aphelinidae) by the predatory ladybeetle *Harmonia axyridis* (Pallas) (Coleoptera: Coccinellidae) in small-scale laboratory trials, a combined application of both natural enemies in larger scale trials improved aphid control in greenhouse roses. In greenhouse sweet pepper infested with FGA, Rocca and Messelink [16] tested the combined use of the predatory brown lacewing *Micromus variegatus* (Fabricius) (Neuroptera: Heherobiidae) and *A. ervi* and found that the combined treatment generally performed no better or only slightly better than the individual treatments. However, they concluded that the combined application might be useful to ensure stable and resilient aphid control. These studies suggest that while IGP of parasitoids via predation of parasitized aphids by predators is common, the activity of the predators and the parasitoids complement each other when used in a large area, so long as the predator does not preferentially and selectively consume parasitized aphids. In our study, *A. baccarum* and *A. ervi* worked in harmony to not only suppress the population growth of FGA but also to eradicate the population. The parasitoid activity was interdependent between the control and the *A. baccarum* treatment and it is manifested in the similar aphid and aphid mummy population dynamics for both treatments (Figure 3A,B). However, the FGA number in the *A. baccarum* treatment decreased more rapidly during the period in which the mites were actually observed, supporting the presence of predation activity by the mites. This biocontrol success is likely due in part to differences in the prey-stage preferences of the two biocontrol agents. Our laboratory trials clearly demonstrated that *A. baccarum* has a strong preference for smaller and younger FGA, whereas *A. ervi* generally parasitizes larger and older FGA [19]. These differences in prey-stage preferences allowed *A. baccarum* and *A. ervi* to share a common resource and resulted in the additive effect observed where species’ interactions are complementary. Another possibility can be interpreted based on the synergistic efficacy reported on a lepidopteran-specialized parasitoid, *Dolichogenidea tasmanica* (Cameron) (Hymenoptera: Braconidae) and *A. baccarum* on brown apple moth larvae, *Epiphyas postvittana* (Walker) (Lepidoptera: Tortricidae) [20]. The moth larvae congregate en masse and make silk webbing for protection that makes them a difficult prey to catch for *A. baccarum*; however, the larvae escape from the protective webbing when the parasitoid attacks, making the larvae more accessible to *A. baccarum*. In the case of the current study, *A. baccarum* prefers to attack isolated aphids than congregated aphids (TS, personal observation). The parasitoid attacks cause aphid groups to disperse or drop off [7], which in turn increases the relative ratio of the isolated aphids to the aphids in congregations, likely improving the predatory efficacy of *A. baccarum*. The relative absence of adult *A. baccarum* and *A. ervi* in the crop between T = 2 and T = 3 weeks can be explained by their life cycles. Adult *A. baccarum* lives about two weeks (T. Saito, unpublished data), as do adult *A. ervi* [21,22]. Between T = 2 and T = 3 weeks, there would have been very few adult *A. baccarum* left but their eggs had been laid. The emergence timing of new generations (the second-generation *A. baccarum* confirmed at T = 4 weeks and the third-generation appeared from T = 7–8 weeks) corresponds with the described life cycle of *A. baccarum* by Cuthbertson et al. [2], which takes four weeks from egg to adult. Our observation from rearing in the laboratory is outlined in Figure 6 and also supports the timing of observed lifecycle events observed in greenhouse sweet pepper.

**Figure 7:**
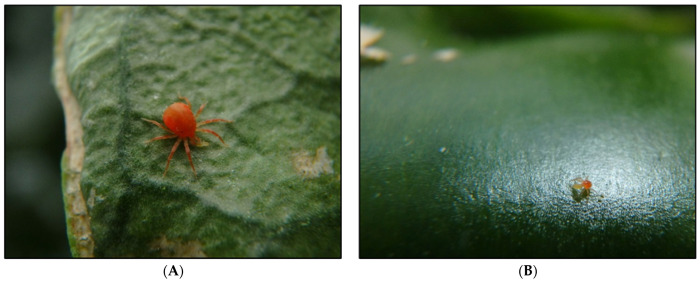
*Anystis baccarum* observed in the greenhouse trial. (**A**) Adult *A. baccarum* consuming a second instar western flower thrips. (**B**) Larval *A. baccarum* consuming a first instar western flower thrips.

Superior FGA biocontrol appears to have resulted in higher fruit yield in the *A. baccarum* treatment (Figure 4A). In addition, *A. baccarum* fed on thrips (Figure 7A,B), especially when there were few live aphids remaining on the plants.

Thrips’ feeding damage on the mature fruits had a high tendency to be less in the *A. baccarum* treatment than the control treatment (Figure 4B). When harvested fruits were examined for signs of damage, *A. baccarum* of different ages were also observed. From fruits harvested at T = 12 weeks, twelve fruits out of total 52 had *A. baccarum* on them and some fruits had as many as five mites on them. It seems that *A. baccarum* likes to hunt and/or hide in the space between the calyx and the fruit itself. This characteristic of *A. baccarum* should be considered carefully when employing the mites in a biocontrol program because the presence of any arthropods (not only pests but also beneficial ones) on finished ornamentals or produce is commonly perceived negatively by consumers. A procedure may be required to eradicate *A. baccarum* before the finished ornamentals or produce are shipped.

At the conclusion of the trial, the coco growbags and plants were checked for *A. baccarum* and their eggs. Although not quantified, several nymphs were found as either mobile predatory stages or quiescent non-predatory chrysalis stages, resting in tight spaces: between the supporting twine and the stem, in cracks in the stem, between the calyx and the fruit, and among the elongated trichomes where the main leaf vein and lateral leaf veins meet on the underside of leaves. Egg masses were found on the coco growbags under the wrapping material. Moreover, several *A. baccarum* nymphs (ca. 10) were found in two of the *A. ervi*-only treatment blocks, highlighting the capacity of *A. baccarum* to spread. Overall, this trial successfully demonstrated that:

- *A. baccarum* can be used with *A. ervi* to control foxglove aphids;
- *A. baccarum* will readily establish on peppers grown in coco growbags; and
- *A. baccarum* provides a robust foundation for a biological control program due to its wide host (prey) range and can complement the activity of a more specialized natural enemy (*A. ervi*).

Mass-rearing *A. baccarum* is challenging but we are currently fine-tuning these techniques. The mass-rearing process is quite space-demanding which can drive up production costs. The application rate employed in this study was a preventative rather than curative rate. Considering the mite is highly cannibalistic and also very voracious against young aphid nymphs, releasing several mites per plant against a small population of aphids does not make sense. A better strategy is to release a limited number of mites and allow them to establish within the crop; this reduces the number of mites needed and maintains lower costs. However, due to its relatively long life cycle, requiring at least two months to build up its population to a curative level, *A. baccarum* is too slow for pests with rapid life cycles and high reproductive rates such as aphids, another reason for using the mite with other aphid management techniques or natural enemies. Based on observations made during maintenance of the mite colonies, a curative rate would be between 5 to 15 mites per plant depending on the pest species, infestation level, and the size of the plant. This makes *A. baccarum* less suited for short-term crops but ideal for biocontrol programs in long-term crops, particularly when used with another natural enemy. Saito and Brownbridge [10] mentioned *A. baccarum* and *Neoseiulus cucumeris* (Oudemans) (Acari: Phytoseiidae) slow-release sachets were complementary in the control of western flower thrips and two-spotted spider mites. Together with the findings of the present study, *A. baccarum* seems to be a useful addition to a biocontrol program if used together with other biocontrol agents that have faster life cycles and preferably with different prey-stage preferences.

## Data Availability

The data presented in this study are available on request from the corresponding author. The data are not publicly available due to the ownership by Vineland Research and Innovation Centre.
